# Prevalence of plasma lipid abnormalities and associated risk factors among Iranian adults based on the findings from STEPs survey 2021

**DOI:** 10.1038/s41598-023-42341-5

**Published:** 2023-09-19

**Authors:** Javad Khanali, Erfan Ghasemi, Mohammad-Mahdi Rashidi, Naser Ahmadi, Seyyed-Hadi Ghamari, Mohammadreza Azangou-Khyavy, Mohammad-Reza Malekpour, Mohsen Abbasi-Kangevari, Seyedeh Melika Hashemi, Mohammadreza Naderian, Negar Rezaei, Arezou Dilmaghani-Marand, Yosef Farzi, Ameneh Kazemi, Moein Yoosefi, Amirali Hajebi, Shahabeddin Rezaei, Sina Azadnajafabad, Nima Fattahi, Maryam Nasserinejad, Elham Abdolhamidi, Rosa Haghshenas, Nazila Rezaei, Shirin Djalalinia, Bagher Larijani, Farshad Farzadfar

**Affiliations:** 1 Non-Communicable Diseases Research Center, Endocrinology and Metabolism Population Sciences Institute, Tehran University of Medical Sciences, Second Floor, No.10, Jalal Al-e-Ahmad Highway, Tehran, 1411713137 Iran; 2 Social Determinants of Health Research Center, Shahid Beheshti University of Medical Sciences, Tehran, Iran; 3 Tehran Heart Center, Cardiovascular Diseases Research Institute, Tehran University of Medical Sciences, Tehran, Iran; 4 Department of Mathematics and Statistics, Memorial University of Newfoundland, St. John’s, NL Canada; 5 Human Nutrition Program, Department of Human Sciences, The Ohio State University, Columbus, OH USA; 6 Department of Internal Medicine, Yale School of Medicine, New Haven, CT USA; 7 Center for Life Course Health ResearchFaculty of Medicine, University of Oulu, Oulu, Finland; 8 Development of Research and Technology Center, Deputy of Research and Technology, Ministry of Health and Medical Education, Tehran, Iran; 9 Endocrinology and Metabolism Research Center, Endocrinology and Metabolism Clinical Sciences Institute, Tehran University of Medical Sciences, Tehran, Iran

**Keywords:** Endocrinology, Risk factors

## Abstract

The study aimed to estimate the prevalence of lipid abnormalities in Iranian adults by demographic characterization, geographical distribution, and associated risk factors using national and sub-national representative samples of the STEPs 2021 survey in Iran. In this population-based household survey, a total of 18,119 individuals aged over 25 years provided blood samples for biochemical analysis. Dyslipidemia was defined by the presence of at least one of the lipid abnormalities of hypertriglyceridemia (≥ 150 mg/dL), hypercholesterolemia (≥ 200 mg/dL), high LDL-C (≥ 130 mg/dL), and low HDL-C (< 50 mg/dL in women, < 40 mg/dL in men), or self-reported use of lipid-lowering medications. Mixed dyslipidemia was characterized as the coexistence of high LDL-C with at least one of the hypertriglyceridemia and low HDL-C. The prevalence of each lipid abnormality was determined by each population strata, and the determinants of abnormal lipid levels were identified using a multiple logistic regression model. The prevalence was 39.7% for hypertriglyceridemia, 21.2% for hypercholesterolemia, 16.4% for high LDL-C, 68.4% for low HDL-C, and 81.0% for dyslipidemia. Hypercholesterolemia and low HDL-C were more prevalent in women, and hypertriglyceridemia was more prevalent in men. The prevalence of dyslipidemia was higher in women (OR = 1.8), obese (OR = 2.8) and overweight (OR = 2.3) persons, those residents in urban areas (OR = 1.1), those with inappropriate physical activity (OR = 1.2), patients with diabetes (OR = 2.7) and hypertension (OR = 1.9), and participants with a history (OR = 1.6) or familial history of CVDs (OR = 1.2). Mixed dyslipidemia prevalence was 13.6% in women and 11.4% in men (P < 0.05). The prevalence of lipid abnormalities was highly heterogeneous among provinces, and East Azarbaijan with 85.3% (81.5–89.1) and Golestan with 68.5% (64.8–72.2) had the highest and lowest prevalence of dyslipidemia, respectively. Although the prevalence of high cholesterol and LDL-C had a descending trend in the 2016–2021 period, the prevalence of dyslipidemia remained unchanged. There are modifiable risk factors associated with dyslipidemia that can be targeted by the primary healthcare system. To modify these risk factors and promote metabolic health in the country, action plans should come to action through a multi-sectoral and collaborative approach.

## Introduction

Metabolic risk factors are considerable public health concerns, and despite measures taken, their global burden has not only increased but also accelerated its growing trend since 1990^1^. Among these risk factors, dyslipidemia has a proven role in atherosclerosis and cardiovascular diseases (CVDs). 4.4 million deaths and 98.6 million disability-adjusted life years (DALYs) in 2019 could be attributed to high LDL cholesterol (LDL-C)^2^. Knowing whether dyslipidemia is declining, stagnating, or even increasing provides governments and international organizations with information on the new health priority and also provides insight into the effectiveness or inadequacy of current efforts^2^. Therefore, repeated cross-sectional and population-based surveys are needed to regularly assess the current circumstance of the risk factor.

Considering the poor metabolic health in the middle-east, tracking dyslipidemia in the region countries, including Iran, would be of special importance^3^. It is shown that high LDL-C contributed to 16.1% of deaths and 7.8% of DALYs caused by non-communicable diseases (NCDs) in the Iranian population in 2019^4^. The high consumption of dietary fats, obesity, physical inactivity, non-adherence to treatment guidelines by patients and physicians, as well as the increasing consumption of carbohydrate-rich foods could predispose the Iranian population to dyslipidemia^5–9^. Meanwhile, the widespread prescribing of statins by general practitioners, restrictions on trans fats in edible oils, and increased public awareness of the risk factor are policies and trends that may decrease dyslipidemia prevalence in Iran^3, 10^.

After recognizing the global need for data on risk factors that drive NCDs, WHO initiated the STEP-wise approach to NCD risk factor surveillance (STEPs) as national household surveys in 2002^11^. Iran is among the few countries in the region that have done regular STEPs surveys on metabolic risk factors, including dyslipidemia^3^. Eight rounds of STEPs surveys have been conducted in Iran; however, only the latest published report, STEPs 2016, presented a complete lipid profile of Iranian adults comprising total cholesterol (TC), triglyceride (TG), LDL-C, and HDL cholesterol (HDL-C)^10^. Therefore, the precise figure of the prevalence of lipid abnormalities in Iran has yet to be clarified. The present study aimed to estimate the prevalence of lipid abnormalities in Iranian adults by demographic characterization, associated risk factors, and geographical distribution. Here, national and sub-national representative samples of the STEPs 2021 survey were analyzed and presented.

## Methods

Based on the STEPwise approach to NCD risk factor surveillance developed by WHO^11^, the STEPs 2021 survey was designed and conducted in Iran with representative samples from urban and rural areas of the country. The details of the procedures and methods of STEPs 2021 have been published^12^, and just a few crucial requirements were discussed here. According to the STEPs established framework, risk factors were assessed in three steps: filling out a questionnaire, obtaining objective information by physical assessment, and collecting participants' blood and urine samples for biochemical analysis (in those aged above 25). All laboratory measurements were performed in the coordinating center in Tehran.

### Sampling and study population

Sampling was done in proportion to the adult population of urban and rural areas of 31 provinces of Iran. Accordingly, a systematic random sampling frame was designed, and 28,821 individuals in 3176 clusters were selected (each including 9 participants). The variables considered in the representative samples were age, gender, area of residence (rural/urban), and province. The number of participants for the third step of the survey was 18,119, including those aged higher than 25 and accepted to participate in lab measurements. The ethics committee of the National Institute for Health Research approved the study protocol (ID: IR.TUMS.NIHR.REC.1398.006), and the study was performed in accordance with the Declaration of Helsinki. The study objectives and methods were clearly explained to all participants along with the fact that participation in the study is voluntary and that refusing to participate will not affect their access to health care.

### Definition and measurement of variables

The participants' wealth index was calculated using factors assessed by the household assets questionnaire, comprising 36 questions asked about various dimensions of participants’ assets. The wealth index was computed by reducing the data dimensions through principal component analysis (PCA), and the first component was assigned to the wealth index and categorized into five quintiles, from the poorest (first quintile) to the richest (fifth quintile)^12^. Smoking was assessed using the transcultural adaption of the STEPS questionnaire. Smoking status was defined as positive for those who are current daily smokers of any tobacco product, including cigarettes, hookah, pipes, smokeless tobacco, and electronic cigarettes^13, 14^. The second version of the global physical activity questionnaire was utilized to assess how many metabolic equivalent (MET) minutes of physical activity were engaged by each participant during a week. Accordingly, appropriate physical activity was defined as having either high (higher or equal to 3000 MET minutes per week) or moderate physical activity (less than 3000, and higher or equal to 600 MET minutes per week), while inappropriate physical activity was defined as having low physical activity (less than 600 MET minute per week)^13, 15^. Those who were consuming at least five servings of fruits and vegetables per day were categorized as those with appropriate fruit and vegetable consumption^13^.

To check for the history of coronary heart disease, patients were asked "Have you ever been told by a physician or health staff that you had a heart attack, chest pain (angina), or have you ever undergone angioplasty (balloon or stent) or coronary artery bypass?". Accordingly, the history of stroke was determined by checking whether the patient was informed by a physician or medical staff to have a stroke. The family history of CVDs was asked using the following question: "Have your father, brother, or son under age 65, or your mother, sister, or daughter under age 55 had a heart attack or stroke, or sudden death?".

Hypertension was defined as SBP ≥ 140 mmHg or DBP ≥ 90 or the use of antihypertensive medications. BMI was categorized accordingly: lower than 18.5 as underweight, above or equal to 18.5 and below 25 as normal, equal to or above 25 but less than 30 as overweight, and equal to or greater than 30 as obese. Fasting plasma glucose, serum TC, HDL-C, and triglyceride (TG) were assessed by the autoanalyzer (Cobas C311 Hitachi High–Technologies Corporation, Japan). Non–HDL-C was calculated by subtracting HDL-C values from TC. LDL-C was estimated using the Friedewald formula. According to the American Diabetes Association definitions, diabetes is defined by fasting plasma glucose of ≥ 126 mg/dL (7 mmol/L) or the use of antihyperglycemic medications^16^. National Cholesterol Education Program, Expert Panel on Detection, Evaluation, and Treatment of High Blood Cholesterol in Adults Treatment Panel III criteria were used to define lipid abnormalities^17, 18^. Plasma lipid abnormalities were defined as follows: Hypertriglyceridemia was defined as serum TG ≥ 150 mg/dL (≥ 1.7 mmol/L). Hypercholesterolemia was defined as TC concentrations of ≥ 200 mg/dL (TC ≥ 5.2 mmol/L). High LDL-C and very high LDL-C were defined as LDL-C concentrations of ≥ 130 mg/dL (LDL-C ≥ 3.4 mmol/L) and ≥ 190 mg/dL (LDL-C ≥ 4.9 mmol/L), respectively. High non-HDL-C was defined as non–HDL-C ≥ 160 mg/dL (non-HDL-C ≥ 4.1 mmol/L). Low HDL-C was defined as serum HDL-C lower than 40 mg/dL (HDL-C < 1.03 mmol/L) in men, and 50 mg/dL (HDL-C < 1.29 mmol/L) in women. Dyslipidemia was characterized by the presence of at least one lipid abnormality of hypertriglyceridemia, hypercholesterolemia, high LDL-C, and low HDL-C or self-reported use of lipid-lowering medications. Mixed dyslipidemia was defined as the coexistence of high LDL-C with at least one of hypertriglyceridemia and low HDL-C.

### Statistical analysis

The prevalence of any lipid abnormality was calculated after applying weights to the samples. The study population was shown to be representative at the national and provincial levels using probability sample tests. Data were summarized by mean and 95% confidence intervals in parentheses [mean (95% confidence interval)]. The chi-square test and One-way ANOVA tests were used to compare categorical and continuous variables between different groups. The multiple logistic regression model was applied to calculate odds ratios (OR) and 95% confidence intervals (95% CI) for the association of socio-demographic, anthropometric, habitual, and medical history variable as independent variables and lipid abnormalities as the outcome. To calculate the adjusted ORs, crude ORs were adjusted for sex, age, and wealth index as covariates. The formula applied for calculating adjusted ORs for each independent variable is as follows, where B0 is the intercept term, X1, X2, and X3 are covariates, and X4 is the independent variable. β terms indicate the coefficients associated with each variable.

$${\text{logit}}\left( {{\text{P}}\left( {{\text{Y}} = {1}} \right)} \right) = \upbeta 0 \, + \, \upbeta {\text{1X1 }} + \, \upbeta {\text{2X2 }} + \, \upbeta {\text{3X3}} + \, \upbeta {\text{4X4}}$$

All statistical analyses were performed with the STATA software version 12 (StataCorp, Texas, USA). Figures were depicted by R. Software version 3.2.1 (Vienna, Austria). Two-tailed P values of 0.05 were considered statistically significant.

### Ethics approval and consent to participate

All participants were informed about the methods and goals of the survey and the fact that participation was voluntary. All participants provided written informed consent. The final dataset was de-identified for analysis. The survey database was accessible only to the primary investigator and the database manager. This study was ethically approved by the National Institute for Health Research's ethical committee (ID: IR.TUMS.NIHR.REC.1398.006), and was performed in accordance with the Declaration of Helsinki. As part of the survey, strict COVID-19 prevention guidelines were implemented during the pandemic for all participants and those involved in the survey/data-gathering step.

## Results

### Population characteristics

Of the total 18,119 participants, 7826 (43.1%) participants were female, and 10,293 (56.8%) participants were male. 24.7% of participants were residents in rural areas (Table 1). Our results showed that 66.6% of the study population were overweight/obese, 57.5% of participants did not have appropriate physical activity, and only 6.2% of them consumed an appropriate amount of fruit and vegetables. 14.1% of the study population were smoking tobacco products daily, including 4.6% of women and 26.0% of men.

**Table 1:** Population characteristics.

Variable	Category	Women	Men	Both
Age category, number Percent (95% CI)	25–39	3454 33.5 (32.2,34.81)	2382 32.29 (30.77,33.8)	5836 32.96 (31.98,33.95)
	40–54	3675 34.77 (33.49,36.06)	2597 32.06 (30.63,33.5)	6272 33.57 (32.61,34.53)
	55–64	1811 17.89 (16.84,18.94)	1501 18.63 (17.42,19.83)	3312 18.22 (17.42,19.01)
	65+	1353 13.83 (12.81,14.85)	1346 17.02 (15.8,18.24)	2699 15.25 (14.46,16.03)
Area of residence, number Percent (95% CI)	Rural	3344 24.49 (23.53,25.46)	2520 24.88 (23.76,26)	5864 24.67 (23.95,25.39)
Wealth index, number Percent (95% CI)	Poor	2190 20.85 (19.68,22.01)	1488 17.71 (16.55,18.86)	3678 18.37 (17.59,19.16)
	2nd quintile	1947 21.76 (20.54,22.99)	1381 18.14 (16.94,19.34)	3328 19.03 (18.21,19.85)
	Middle	1944 18.12 (17.13,19.11)	1744 19.93 (18.79,21.08)	3688 17.95 (17.24,18.66)
	4th quintile	1813 19.43 (18.33,20.53)	1658 21.23 (20.01,22.45)	3471 19.18 (18.4,19.96)
	Rich	1563 19.84 (18.6,21.08)	1419 22.99 (21.42,24.56)	2982 20.16 (19.22,21.1)
BMI category, number Percent (95% CI)	Underweight	217 1.84 (1.55,2.13)	248 2.69 (2.26,3.12)	465 2.2 (1.96,2.45)
	Normal weight	2636 25.32 (24.13,26.52)	2994 37.67 (36.15,39.19)	5630 30.63 (29.68,31.57)
	Overweight	3925 38.96 (37.6,40.32)	3131 41.02 (39.45,42.58)	7056 39.64 (38.62,40.67)
	Obese	3468 33.88 (32.58,35.18)	1419 18.63 (17.35,19.9)	4887 26.95 (26.03,27.87)
Appropriate fruit and vegetable consumption, number Percent (95% CI)	Yes	755 6.94 (6.3,7.58)	434 5.41 (4.72,6.1)	1189 6.25 (5.79,6.72)
Inappropriate physical activity, number Percent (95% CI)	Yes	5530 57.51 (56.14,58.88)	2590 41.8 (40.05,43.54)	8120 46.66 (45.63,47.7)
Smoking (current daily smoking), number Percent (95% CI)	Yes	476 4.61 (4.09,5.19)	1993 26.03 (24.62,27.48)	2469 14.11 (13.38,14.87)
History of diabetes, number Percent (95% CI)	Yes	1456 14.71 (13.76,15.65)	987 13.45 (12.32,14.57)	2443 14.13 (13.41,14.86)
History of hypertension, number Percent (95% CI)	Yes	3847 36.84 (35.5,38.17)	2807 34.71 (33.2,36.22)	6654 35.75 (34.75,36.75)
History of coronary heart disease, number Percent (95% CI)	Yes	637 6.42 (5.72,7.11)	718 9.42 (8.5,10.33)	1355 7.73 (7.17,8.29)
History of stroke, number Percent (95% CI)	Yes	128 1.16 (0.9,1.42)	149 1.86 (1.45,2.27)	277 1.47 (1.23,1.7)
Familial history of cardiovascular disease, number Percent (95% CI)	Yes	1451 15.14 (14.14,16.13)	850 11.54 (10.49,12.58)	2301 13.2 (12.49,13.9)

Regarding past medical and family history, 14.1% of participants were diabetic and 35.7% had hypertension. 7.7% and 1.5% of participants mentioned they had a history of coronary heart disease and stroke, respectively. 13.2% of participants mentioned a family history of CVD.

### Prevalence of lipid abnormalities in different population strata

The prevalence of dyslipidemia was 81.0% (80.2–81.9) among the Iranian adult population, affecting 84.4% (83.4–85.4) of women and 75.7% (74.4–77.1) of men (Table 2). Of the total female adult population, 35.5% (34.2–36.9) had hypertriglyceridemia, 23.0% (21.8–24.2) had hypercholesterolemia, 17.1% (16.0–18.3) had high LDL-C, and 72.7% (71.5–73.9) had low HDL-C. Of the total male adult population, 43.4% (41.9–45.0) had hypertriglyceridemia, 18.9% (17.7–20.2) had hypercholesterolemia, 15.4% (14.2–16.5) had high LDL-C, and 62.0% (60.5–63.5) had low HDL-C. Accordingly, hypercholesterolemia and low HDL-C were more prevalent in women, and hypertriglyceridemia was more prevalent in men. 19.4% (18.5–20.3) of the population had high non-HDL-C, and the abnormality had a similar prevalence among men and women. The age group of 55–64 had the highest prevalence of dyslipidemia, which was significantly higher than the 25–39 age groups (83.3% vs. 77.0%; P < 0.05) (Table 2). Compared to the reference age group (25–39), other age groups had higher odds of having hypertriglyceridemia, hypercholesterolemia, and high LDL-C; however, those older than 55 had lower odds of having low HDL-C (Table 3). Supplementary Table S1 shows the value of serum lipids in different centiles for different age strata.

**Table 2:** Prevalence of lipid abnormalities according to study population strata.

Variable	Category	Hypertriglyceridemia Percent. (95% CI)	Hypercholesterolemia Percent. (95% CI)	High LDL-C Percent. (95% CI)	Very High LDL-C Percent. (95% CI)	High non–HDL-C Percent. (95% CI)	Low HDL-C Percent. (95% CI)	Dyslipidemia Percent. (95% CI)
All participants	–	39.7 (38.6,40.79)	21.24 (20.32,22.17)	16.42 (15.56,17.29)	0.74 (0.52,0.95)	19.43 (18.54,20.33)	68.42 (67.41,69.42)	81.02 (80.17,81.87)
Sex	Women	35.54 (34.21,36.87)	23.04 (21.84,24.23)	17.15 (16.04,18.27)	0.76 (0.58,0.94)	19.03 (17.9,20.15)	72.68 (71.48,73.89)	84.41 (83.43,85.4)
	Men	43.45 (41.87,45.04)	18.94 (17.69,20.18)	15.36 (14.19,16.53)	0.74 (0.35,1.12)	19.68 (18.42,20.94)	62 (60.48,63.51)	75.73 (74.4,77.06)
Age	25–39	32.52 (30.71,34.33)	12.14 (10.95,13.33)	9.43 (8.28,10.58)	0.33 (0.18,0.49)	11.76 (10.61,12.92)	70.63 (69.02,72.24)	77.05 (75.56,78.55)
	40–54	42.79 (41.1,44.48)	24.65 (23.17,26.13)	18.44 (17.11,19.76)	0.89 (0.39,1.39)	23.02 (21.56,24.48)	69.37 (67.79,70.96)	82.01 (80.67,83.34)
	55–64	44.61 (42.23,46.98)	29 (26.84,31.15)	22.36 (20.35,24.38)	0.93 (0.58,1.29)	25.01 (23,27.03)	65.09 (62.8,67.37)	83.29 (81.42,85.15)
	65+	38.34 (35.44,41.25)	24.02 (21.3,26.74)	19.61 (16.94,22.27)	1.14 (0.71,1.57)	20.7 (18.03,23.37)	62.36 (59.67,65.05)	81.7 (79.68,83.73)
Area	Rural	35.67 (34.2,37.13)	21.35 (20.08,22.62)	17.19 (16.02,18.36)	0.83 (0.57,1.09)	19.46 (18.22,20.69)	66.68 (65.25,68.11)	78.8 (77.56,80.04)
	Urban	40.16 (38.88,41.43)	21.17 (20.1,22.25)	16.09 (15.08,17.09)	0.73 (0.48,0.97)	19.27 (18.23,20.3)	68.35 (67.18,69.53)	81.14 (80.15,82.13)
Wealth index	Poor	35.95 (33.62,38.28)	22.04 (19.82,24.27)	17.08 (14.93,19.23)	0.95 (0.6,1.31)	19.09 (17,21.17)	67.75 (65.65,69.85)	79.69 (77.84,81.54)
	2nd quintile	39.85 (37.45,42.25)	19.21 (17.5,20.93)	14.61 (13.1,16.12)	0.76 (0.46,1.07)	17.68 (16.03,19.33)	68.71 (66.51,70.92)	80.96 (79.07,82.84)
	Middle	39.56 (37.44,41.68)	20.03 (18.28,21.78)	15.76 (14.12,17.4)	0.72 (0.41,1.04)	19.3 (17.55,21.04)	68.15 (66.24,70.06)	79.98 (78.36,81.59)
	4th quintile	40.26 (38.1,42.43)	22.05 (20.24,23.87)	16 (14.45,17.54)	0.5 (0.26,0.74)	20.05 (18.3,21.81)	67.01 (64.89,69.13)	79.95 (78.03,81.87)
	Rich	40.48 (37.74,43.21)	22.08 (19.77,24.39)	17.94 (15.68,20.2)	0.96 (0.16,1.76)	20.51 (18.22,22.79)	66.91 (64.36,69.45)	81.1 (79.09,83.12)
BMI Category	Underweight	6.15 (3.93,8.37)	8.76 (6.01,11.51)	8.81 (5.87,11.74)	0.4 (-0.09,0.89)	5.21 (3.23,7.18)	39.05 (33.57,44.53)	48.52 (42.86,54.17)
	Normal weight	27.38 (25.67,29.08)	17.36 (15.88,18.84)	14.45 (13.08,15.83)	0.38 (0.22,0.55)	15.05 (13.63,16.47)	57.67 (55.85,59.48)	70.48 (68.84,72.11)
	Overweight	41.82 (40.16,43.48)	22.84 (21.44,24.24)	17.32 (16.04,18.61)	1.04 (0.6,1.49)	20.93 (19.62,22.25)	71.7 (70.21,73.18)	84.81 (83.58,86.05)
	Obese	50.67 (48.67,52.68)	23.91 (22.16,25.66)	17.5 (15.83,19.17)	0.78 (0.53,1.04)	22.7 (20.93,24.47)	76.38 (74.71,78.04)	88.17 (86.84,89.49)
Fruit and vegetable consumption	Inappropriate	39.01 (37.95,40.08)	21.24 (20.34,22.14)	16.34 (15.5,17.18)	0.76 (0.55,0.97)	19.29 (18.42,20.16)	67.77 (66.78,68.76)	80.35 (79.51,81.19)
	Appropriate	40.01 (36.27,43.75)	21.03 (17.83,24.23)	16.74 (13.71,19.78)	0.64 (0.18,1.09)	19.83 (16.82,22.83)	70.15 (66.58,73.71)	83.36 (80.65,86.06)
Physical activity	Inappropriate	40.24 (38.71,41.78)	21.99 (20.64,23.34)	16.9 (15.63,18.18)	0.74 (0.37,1.1)	19.78 (18.47,21.09)	70.58 (69.22,71.94)	83.31 (82.22,84.4)
	appropriate	38.55 (37.01,40.09)	20.95 (19.7,22.2)	16.07 (14.91,17.22)	0.76 (0.56,0.96)	19.08 (17.88,20.28)	66.91 (65.45,68.38)	79.44 (78.16,80.71)
Smoking	No	38.09 (36.99,39.18)	21.67 (20.73,22.61)	16.54 (15.66,17.42)	0.71 (0.57,0.85)	19.17 (18.27,20.07)	67.85 (66.81,68.88)	80.84 (79.97,81.72)
	Yes	44.75 (41.82,47.68)	18.35 (16.17,20.53)	15.11 (13.04,17.17)	1 (-0.11,2.11)	20.05 (17.77,22.33)	68.62 (66.14,71.09)	78.82 (76.7,80.94)
Diabetes	No	35.9 (34.8,37)	21.07 (20.13,22.02)	16.67 (15.78,17.56)	0.73 (0.5,0.95)	18.95 (18.05,19.86)	66.52 (65.48,67.56)	78.76 (77.86,79.66)
	Yes	58.18 (55.43,60.93)	22.05 (19.83,24.27)	14.43 (12.58,16.27)	0.9 (0.52,1.28)	21.47 (19.28,23.67)	76.63 (74.31,78.94)	91.38 (89.71,93.04)
Hypertension	No	34.16 (32.92,35.39)	18.89 (17.86,19.93)	14.82 (13.86,15.78)	0.58 (0.31,0.86)	16.99 (16,17.97)	66.07 (64.86,67.29)	77.12 (76.05,78.19)
	Yes	47.61 (45.85,49.37)	25.21 (23.66,26.75)	19.03 (17.58,20.49)	1.06 (0.79,1.32)	23.36 (21.83,24.88)	71.3 (69.78,72.82)	86.58 (85.41,87.76)
History of coronary heart disease	No	38.71 (37.64,39.78)	21.55 (20.64,22.46)	16.66 (15.8,17.51)	0.76 (0.55,0.97)	19.66 (18.78,20.54)	67.58 (66.59,68.58)	80.04 (79.19,80.89)
	Yes	42.76 (39.01,46.51)	16.99 (14.29,19.68)	12.36 (10.04,14.68)	0.64 (0.19,1.08)	14.93 (12.39,17.47)	72.2 (68.89,75.52)	86.42 (83.8,89.03)
History of stroke	No	39.02 (37.98,40.05)	21.18 (20.3,22.05)	16.31 (15.5,17.13)	0.76 (0.56,0.96)	19.28 (18.43,20.12)	67.95 (66.99,68.91)	80.46 (79.64,81.27)
	Yes	39.7 (31.8,47.59)	22.33 (15.16,29.5)	17.01 (10.86,23.16)	0.22 (-0.09,0.53)	20.35 (13.79,26.92)	67.72 (60.29,75.15)	85.92 (80.11,91.74)
Familial history of CVD	No	38.22 (37.11,39.33)	20.91 (19.97,21.84)	16.12 (15.24,17)	0.61 (0.48,0.74)	18.74 (17.84,19.64)	67.26 (66.22,68.31)	79.95 (79.06,80.85)
	Yes	43.86 (40.96,46.76)	22.63 (20.2,25.07)	17.41 (15.13,19.69)	1.54 (0.32,2.77)	22.02 (19.57,24.48)	71.6 (69.12,74.08)	83.76 (81.69,85.83)

**Table 3:** Lipid abnormalities in association with the study population characteristics.

Variable (reference group)	Category	Hypertriglyceridemia		Hypercholesterolemia		High LDL-C		Low HDL-C		Dyslipidemia	
		Crude OR (95% CI)	Adjusted OR (95% CI)	Crude OR (95% CI)	Adjusted OR (95% CI)	Crude OR (95% CI)	Adjusted OR (95% CI)	Crude OR (95% CI)	Adjusted OR (95% CI)	Crude OR (95% CI)	Adjusted OR (95% CI)
Sex (female)	Male	1.39 (1.28,1.52)	1.38 (1.26,1.51)	0.78 (0.70,0.87)	0.77 (0.69,0.86)	0.88 (0.78,0.99)	0.87 (0.77,0.98)	0.61 (0.56,0.67)	0.62 (0.56,0.68)	0.588 (0.52,0.64)	0.56 (0.50,0.63)
Age (25–39 y)	40–54	1.55 (1.39,1.73)	1.56 (1.40,1.74)	2.37 (2.06,2.71)	2.34 (2.03,2.70)	2.17 (1.85,2.55)	2.13 (1.80,2.51)	0.94 (0.85,1.05)	0.95 (0.85,1.06)	1.36 (1.20,1.54)	1.37 (1.21,1.56)
	55–64	1.67 (1.47,1.90)	1.64 (1.44,1.87)	2.95 (2.54,3.44)	2.94 (2.51,3.45)	2.77 (2.32,3.30)	2.75 (2.29,3.30)	0.77 (0.68,0.88)	0.78 (0.69,0.89)	1.48 (1.27,1.74)	1.51 (1.28,1.78)
	> 65	1.29 (1.11,1.50)	1.28 (1.10,1.49)	2.29 (1.90,2.75)	2.31 (1.91,2.81)	2.34 (1.89,2.91)	2.34 (1.87,2.93)	0.69 (0.60,0.79)	0.71 (0.62,0.82)	1.30 (1.13,1.56)	1.38 (1.17,1.63)
Area (rural)	Urban	1.21 (1.11,1.32)	1.21 (1.10,1.32)	0.99 (0.90,1.09)	0.97 (0.87,1.085)	0.92 (0.83,1.03)	0.89 (0.78,1.01)	1.08 (0.99,1.174)	1.13 (1.03,1.24)	1.16 (1.05,1.28)	1.14 (1.02,1.27)
Wealth index (poor)	2nd quintile	1.18 (1.02,1.36)	1.17 (1.02,1.35)	0.841 (0.71,1.00)	0.84 (0.71,0.99)	0.83 (0.68,1.01)	0.83 (0.69,1.01)	1.05 (0.91,1.20)	1.03 (0.89,1.19)	1.08 (0.92,1.28)	1.08 (0.91,1.28)
	Middle	1.17 (1.02,1.33)	1.13 (0.99,1.29)	0.89 (0.75,1.05)	0.91 (0.77,1.08)	0.91 (0.75,1.10)	0.94 (0.77,1.14)	1.02 (0.89,1.16)	1.02 (0.89,1.16)	1.02 (0.87,1.18)	1.06 (0.91,1.24)
	4th quintile	1.20 (1.05,1.37)	1.17 (1.02,1.33)	1.00 (0.85,1.18)	1.04 (0.88,1.23)	0.92 (0.76,1.12)	0.96 (0.79,1.16)	0.97 (0.84,1.11)	0.96 (0.84,1.11)	1.02 (0.86,1.20)	1.06 (0.9,1.26)
	Rich	1.21 (1.04,1.41)	1.16 (0.99,1.35)	1.00 (0.83,1.21)	1.03 (0.86,1.24)	1.06 (0.86,1.32)	1.10 (0.89,1.36)	0.96 (0.83,1.12)	0.96 (0.83,1.12)	1.09 (0.92,1.30)	1.15 (0.96,1.37)
BMI category (underweight)	Normal weight	5.75 (3.88,8.53)	5.44 (3.63,8.15)	2.19 (1.53,3.13)	2.06 (1.43,2.96)	1.75 (1.19,2.56)	1.69 (1.14,2.51)	2.13 (1.67,2.71)	2.28 (1.75,2.96)	2.53 (1.99,3.22)	2.54 (1.98,3.26)
	Overweight	10.97 (7.42,16.21)	10.64 (7.11,15.93)	3.08 (2.17,4.39)	2.63 (1.83,3.76)	2.17 (1.49,3.16)	1.94 (1.32,2.87)	3.95 (3.11,5.03)	4.30 (3.29,5.61)	5.93 (4.64,7.58)	5.73 (4.42,7.42)
	Obese	15.68 (10.58,23.22)	16.37 (10.88,24.63)	3.27 (2.29,4.68)	2.51 (1.73,3.63)	2.20 (1.50,3.22)	1.81 (1.20,2.72)	5.05 (3.94,6.46)	5.32 (4.05,7.001)	7.91 (6.10,10.25)	7.02 (5.34,9.22)
Fruit and vegetable consumption (inappropriate)	appropriate	1.04 (0.89,1.23)	1.02 (0.86,1.22)	0.99 (0.81,1.21)	0.92 (0.75,1.14)	1.03 (0.82,1.29)	0.97 (0.77,1.22)	1.12 (0.94,1.33)	1.08 (0.89,1.30)	1.22 (1.00,1.50)	1.18 (0.95,1.47)
Physical activity (appropriate)	Inappropriate	1.07 (0.98,1.18)	1.15 (1.04,1.26)	1.06 (0.95,1.19)	0.99 (0.88,1.11)	1.06 (0.94,1.20)	1.00 (0.88,1.14)	1.19 (1.08,1.30)	1.13 (1.03,1.25)	1.29 (1.16,1.44)	1.19 (1.06,1.33)
Smoking (no)	Yes	1.32 (1.16,1.50)	1.19 (1.04,1.37)	0.81 (0.69,0.95)	0.93 (0.78,1.09)	0.90 (0.76,1.07)	0.98 (0.82,1.18)	1.04 (0.91,1.17)	1.27 (1.11,1.45)	0.88 (0.77,1.01)	1.14 (0.98,1.33)
Diabetes (no)	Yes	2.48 (2.20,2.81)	2.39 (2.09,2.73)	1.06 (0.92,1.22)	0.78 (0.67,0.92)	0.84 (0.72,0.99)	0.61 (0.51,0.73)	1.65 (1.44,1.89)	1.90 (1.63,2.20)	2.86 (2.30,3.55)	2.69 (2.14,3.38)
Hypertension (no)	Yes	1.75 (1.60,1.92)	1.84 (1.65,2.04)	1.45 (1.30,1.61)	1.08 (0.95,1.23)	1.35 (1.20,1.53)	1.00 (0.86,1.16)	1.28 (1.16,1.40)	1.57 (1.40,1.74)	1.91 (1.70,2.15)	1.91 (1.67,2.18)
History of coronary heart disease (no)	Yes	1.18 (1.01,1.39)	1.05 (0.88,1.25)	0.74 (0.61,0.91)	0.56 (0.45,0.70)	0.71 (0.56,0.88)	0.52 (0.41,0.66)	1.25 (1.05,1.48)	1.58 (1.31,1.91)	1.59 (1.26,1.99)	1.61 (1.26,2.06)
History of stroke (no)	Yes	1.03 (0.74,1.43)	0.91 (0.64,1.28)	1.07 (0.71,1.62)	0.89 (0.58,1.39)	1.05 (0.68,1.63)	0.86 (0.54,1.36)	1.00 (0.70,1.39)	1.19 (0.84,1.70)	1.48 (0.91,2.40)	1.43 (0.87,2.34)
Familial history of CVD (no)	Yes	1.26 (1.11,1.43)	1.26 (1.11,1.44)	1.11 (0.95,1.29)	1.06 (0.90,1.24)	1.10 (0.92,1.30)	1.06 (0.88,1.26)	1.23 (1.08,1.40)	1.18 (1.03,1.36)	1.29 (1.10,1.52)	1.20 (1.01,1.42)

Adjusted for sex, age, and wealth index.

Dyslipidemia was more prevalent in obese people (88.1%; 95% CI 86.8–89.5), in residents of urban areas (81.1%, 95% CI 80.1–82.1), in those with inappropriate physical activity (83.3%; 95% CI 82.2–84.4), and patients with diabetes (91.4%, 95% CI 89.7–93.0) and hypertension (86.6%, 95% CI 85.4–87.8) (Table 2). Compared to underweight people, those who were normal weight, overweight, and obese had 2.5 (2.0–3.3), 5.7 (4.4–7.4), and 7.0 (5.3–9.2) times higher odds of having dyslipidemia, respectively (Table 3). Residence in urban areas, Inappropriate physical activity, diabetes, and hypertension also increased the odds of having dyslipidemia by 1.1 (1–1.3), 1.2 (1.1–1.3), 2.7 (2.1–3.4), and 1.9 (1.7–2.2) times.

Patients with a history of coronary heart disease and stroke also showed higher prevalence and odds for dyslipidemia. The prevalence in patients with a history of coronary heart disease was 86.4% (83.8–89.0) and the odds ratio was 1.6 (1.3–2.1) (Table 3). Dyslipidemia prevalence in patients with a history of stroke was also higher (85.9% in participants with positive history vs. 80.5 in participants with negative history); however, the difference was not significant due to the small number of cases (n = 277). Those with familial history of CVDs also showed a slightly higher prevalence of dyslipidemia with an odds ratio of 1.2 (1.0–1.4). People with a different wealth index, fruit and vegetable consumption, and smoking status did not show any difference in the prevalence of dyslipidemia, albeit it was not the case for all kinds of lipid abnormalities (Tables 2, 3). The mean value of serum lipids in each population strata is demonstrated in Supplementary Table S2.

### Mixed lipid abnormalities

Mixed dyslipidemia which was defined as the coexistence of high LDL-C with at least one of the hypertriglyceridemia and low HDL-C, was prevalent in 13.6% of women and 11.4% of men (Table 4). In comparison, the isolated high LDL-C had a low prevalence, 3.6% (3.1–4.1) in women and 3.9% (3.4–4.5) in men. Among different mixed dyslipidemias, having high LDL-C and TG and Low HDL-C combined was the most prevalent in both sexes, with a prevalence of 7.4% (6.5–8.2) in women and 6.5% (5.7–7.4) in men.

**Table 4:** Prevalence of mixed lipid abnormalities and their association with the study population characteristics.

Variable	Category	High LDL-C alone			High LDL-C and high TG			High LDL-C and low HDL-C			High LDL-C and TG and low HDL-C		
		Prevalence percent. (95% CI)	Crude OR (95% CI)	Adjusted OR (95% CI)	Prevalence percent. (95% CI)	Crude OR (95% CI)	Adjusted OR (95% CI)	Prevalence percent. (95% CI)	Crude OR (95% CI)	Adjusted OR (95% CI)	Prevalence percent. (95% CI)	Crude OR (95% CI)	Adjusted OR (95% CI)
Sex	Women	3.59 (3.08,4.1)	1	1	1.33 (1.03,1.62)	1	1	4.85 (4.22,5.49)	1	1	7.38 (6.55,8.22)	1	1
	Men	3.93 (3.39,4.47)	1.10 (0.90,1.3)	1.05 (0.84,1.30)	2.50 (1.95,3.04)	1.90 (1.39,2.61)	1.78 (1.31,2.42)	2.40 (1.92,2.88)	0.48 (0.38,0.62)	0.50 (0.38,0.64)	6.52 (5.66,7.38)	0.87 (0.73,1.05)	0.88 (0.73,1.06)
Age	25–39	1.79 (1.37,2.21)	1	1	0.95 (0.63,1.27)	1	1	2.31 (1.63,2.99)	1	1	4.38 (3.55,5.20)	1	1
	40–54	4.16 (3.52,4.80)	2.38 (1.78,3.18)	2.39 (1.77,3.21)	2.11 (1.61,2.60)	2.24 (1.48,3.40)	2.19 (1.44,3.33)	4.41 (3.72,5.09)	1.95 (1.38,2.74)	1.86 (1.30,2.66)	7.76 (6.81,8.71)	1.84 (1.45,2.33)	1.81 (1.41,2.32)
	55–64	5.55 (4.35,6.75)	3.22 (2.31,4.49)	3.21 (2.29,4.50)	2.74 (2.04,3.43)	2.94 (1.91,4.51)	2.75 (1.78,4.24)	4.75 (3.64,5.86)	2.11 (1.43,3.11)	2.17 (1.45,3.26)	9.32 (7.99,10.64)	2.24 (1.75,2.89)	2.21 (1.70,2.87)
	> 65	4.89 (3.89,5.88)	2.82 (2.04,3.89)	2.72 (1.94,3.82)	2.15 (1.02,3.29)	2.30 (1.22,4.34)	2.20 (1.14,4.26)	4.33 (3.27,5.39)	1.91 (1.29,2.84)	1.87 (1.21,2.88)	8.24 (5.93,10.55)	1.96 (1.36,2.82)	2.05 (1.42,2.95)
Area	Rural	4.35 (3.73,4.96)	1	1	1.67 (1.28,2.06)	1	1	3.72 (3.15,4.28)	1	1	7.45 (6.61,8.29)	1	1
	Urban	3.54 (3.09,3.99)	0.81 (0.66,0.98)	0.75 (0.61,0.92)	1.90 (1.54,2.27)	1.14 (0.84,1.55)	1.03 (0.76,1.39)	3.78 (3.27,4.30)	1.02 (0.82,1.26)	1.06 (0.82,1.37)	6.85 (6.11,7.60)	0.91 (0.77,1.08)	0.89 (0.73,1.08)
Wealth index	Poor	3.77 (3.03,4.52)	1	1	1.67 (1.21,2.14)	1	1	4.92 (3.63,6.21)	1	1	6.70 (4.95,8.46)	1	1
	2nd quintile	3.55 (2.80,4.30)	0.94 (0.69,1.27)	0.94 (0.70,1.28)	1.81 (1.18,2.44)	1.08 (0.69,1.70)	1.08 (0.69,1.70)	3.26 (2.50,4.01)	0.65 (0.45,0.94)	0.65 (0.45,0.94)	6.00 (5.03,6.96)	0.89 (0.64,1.23)	0.89 (0.65,1.23)
	Middle	3.75 (2.93,4.58)	0.99 (0.73,1.35)	1.02 (0.75,1.40)	1.71 (1.20,2.23)	1.02 (0.67,1.55)	0.99 (0.65,1.52)	3.42 (2.75,4.10)	0.68 (0.49,0.96)	0.73 (0.51,1.03)	6.87 (5.57,8.16)	1.03 (0.73,1.45)	1.06 (0.76,1.48)
	4th quintile	3.62 (2.89,4.35)	0.96 (0.71,1.28)	0.99 (0.73,1.33)	2.18 (1.58,2.79)	1.31 (0.88,1.96)	1.28 (0.85,1.94)	2.91 (2.20,3.62)	0.58 (0.40,0.84)	0.61 (0.41,0.91)	7.28 (6.16,8.39)	1.09 (0.79,1.51)	1.14 (0.84,1.54)
	Rich	4.16 (3.03,5.29)	1.11 (0.78,1.57)	1.13 (0.80,1.60)	2.18 (1.17,3.18)	1.31 (0.75,2.27)	1.25 (0.68,2.28)	3.81 (2.75,4.87)	0.77 (0.51,1.14)	0.81 (0.54,1.22)	7.79 (6.16,9.41)	1.17 (0.82,1.68)	1.22 (0.87,1.70)
BMI category	Underweight	4.48 (2.44,6.52)	1	1	0.77 (− 0.02,1.56)	1	1	3.14 (1.13,5.15)	1	1	0.42 (− 0.02,0.86)	1	1
	Normal weight	4.28 (3.58,4.98)	0.95 (0.57,1.58)	0.81 (0.49,1.35)	2.05 (1.36,2.73)	2.69 (0.91,7.97)	2.42 (0.82,7.12)	3.60 (2.87,4.32)	1.15 (0.58,2.31)	1.34 (0.61,2.95)	4.53 (3.69,5.36)	11.29 (3.86,33.02)	10.19 (3.47,29.90)
	Overweight	4.11 (3.46,4.76)	0.91 (0.55,1.51)	0.72 (0.43,1.19)	2.02 (1.60,2.45)	2.66 (0.93,7.61)	2.30 (0.80,6.62)	3.79 (3.07,4.51)	1.22 (0.61,2.43)	1.32 (0.59,2.9)	7.40 (6.52,8.28)	19.02 (6.56,55.10)	15.93 (5.47,46.41)
	Obese	2.55 (2.00,3.11)	0.56 (0.33,0.94)	0.41 (0.23,0.71)	1.48 (1.07,1.90)	1.94 (0.67,5.65)	1.74 (0.59,5.15)	3.99 (3.28,4.71)	1.28 (0.65,2.55)	1.16 (0.52,2.57)	9.46 (7.98,10.94)	24.88 (8.53,72.52)	20.14 (6.79,59.75)
Fruit and vegetable consumption	Inappropriate	3.73 (3.36,4.10)	1	1	2.19 (1.21,3.18)	1	1	2.86 (1.76,3.97)	1	1	7.65 (5.56,9.75)	1	1
	Appropriate	4.02 (2.01,6.03)	1.08 (0.63,1.84)	1.11 (0.66,1.87)	1.83 (1.52,2.13)	1.21 (0.74,1.97)	1.22 (0.73,2.05)	3.83 (3.40,4.27)	0.74 (0.49,1.12)	0.73 (0.47,1.14)	6.95 (6.32,7.57)	1.11 (0.81,1.52)	0.96 (0.68,1.34)
Physical activity	Appropriate	3.95 (3.35,4.55)	1	1	2.07 (1.55,2.58)	1	1	3.81 (3.21,4.42)	1	1	6.23 (5.49,6.97)	1	1
	Inappropriate	3.26 (2.77,3.74)	0.82 (0.66,1.02)	0.78 (0.62,0.99)	1.60 (1.24,1.95)	0.77 (0.55,1.08)	0.84 (0.59,1.19)	4.02 (3.37,4.67)	1.06 (0.83,1.34)	0.91 (0.70,1.18)	8.02 (6.98,9.06)	1.31 (1.09,1.59)	1.26 (1.04,1.54)
Smoking	No	3.89 (3.48,4.30)	1	1	1.85 (1.53,2.18)	1	1	3.84 (3.39,4.30)	1	1	6.94 (6.3,7.59)	1	1
	Yes	2.87 (2.03,3.70)	0.73 (0.53,1.00)	0.71 (0.51,0.99)	1.68 (1.07,2.30)	0.91 (0.60,1.37)	0.72 (0.47,1.09)	3.33 (2.32,4.34)	0.86 (0.62,1.20)	1.20 (0.84,1.71)	7.23 (5.60,8.85)	1.04 (0.80,1.36)	1.18 (0.90,1.54)
Diabetes	No	4.09 (3.67,4.51)	1	1	1.77 (1.46,2.08)	1	1	4.09 (3.62,4.56)	1	1	6.71 (6.06,7.37)	1	1
	Yes	1.63 (1.02,2.24)	0.39 (0.26,0.57)	0.28 (0.18,0.43)	2.15 (1.36,2.95)	1.22 (0.80,1.86)	0.94 (0.59,1.50)	1.85 (1.27,2.42)	0.44 (0.31,0.62)	0.35 (0.24,0.50)	8.79 (7.27,10.32)	1.34 (1.08,1.66)	1.04 (0.82,1.33)
Hypertension	No	3.73 (3.27,4.20)	1	1	1.66 (1.28,2.03)	1	1	3.72 (3.17,4.26)	1	1	5.71 (5.08,6.34)	1	1
	Yes	3.79 (3.17,4.40)	1.01 (0.82,1.25)	0.70 (0.55,0.90)	2.15 (1.70,2.60)	1.30 (0.95,1.79)	1.03 (0.68,1.54)	3.87 (3.25,4.48)	1.04 (0.83,1.30)	0.71 (0.54,0.92)	9.23 (8.00,10.45)	1.68 (1.39,2.02)	1.44 (1.13,1.84)
History of coronary heart disease	No	3.83 (3.45,4.22)	1	1	1.89 (1.58,2.20)	1	1	3.85 (3.41,4.29)	1	1	7.08 (6.44,7.71)	1	1
	Yes	2.72 (1.37,4.07)	0.70 (0.42,1.18)	0.52 (0.30,0.89)	1.11 (0.47,1.75)	0.58 (0.32,1.07)	0.41 (0.22,0.79)	2.67 (1.70,3.64)	0.68 (0.46,1.01)	0.57 (0.38,0.87)	5.86 (4.25,7.48)	0.82 (0.60,1.11)	0.63 (0.45,0.89)
History of stroke	No	3.75 (3.37,4.12)	1	1	1.84 (1.55,2.13)	1	1	3.73 (3.32,4.14)	1	1	6.99 (6.39,7.6)	1	1
	Yes	3.84 (1.61,6.06)	1.02 (0.56,1.89)	0.73 (0.38,1.39)	1.3 (− 0.06,2.65)	0.70 (0.24,2.05)	0.53 (0.18,1.58)	5.71 (0.63,10.79)	1.56 (0.60,4.04)	1.51 (0.56,4.06)	6.17 (2.89,9.45)	0.87 (0.49,1.55)	0.77 (0.42,1.38)
Familial history of CVD	No	3.78 (3.37,4.18)	1	1	1.85 (1.52,2.17)	1	1	3.7 (3.25,4.15)	1	1	6.79 (6.15,7.44)	1	1
	Yes	3.54 (2.56,4.51)	0.93 (0.69,1.27)	0.92 (0.67,1.27)	1.62 (0.97,2.26)	0.87 (0.56,1.36)	0.83 (0.52,1.32)	4.25 (3.08,5.41)	1.15 (0.84,1.58)	1.13 (0.81,1.56)	8.01 (6.21,9.81)	1.19 (0.92,1.56)	1.14 (0.86,1.50)

Those with higher age had higher odds of having all types of mixed dyslipidemias (Table 4). Moreover, higher BMI scores were associated with having high LDL-C and TG and Low HDL-C combined (OR = 10.2 for normal weight, 15.9 for overweight, and 20.1 for obese participants). The same pattern was shown in those with low physical activity (OR = 1.3; 95% CI 1.0–1.5) and hypertension (OR = 1.4; 95% CI 1.1–1.8). On the other side, a history of coronary heart disease was negatively associated with all types of mixed dyslipidemias. Having diabetes was also negatively associated with having high LDL-C and Low HDL-C combined (OR = 0.3; 95% CI 0.2–0.5).

### Prevalence of lipid abnormalities by province

**Figure 1:**
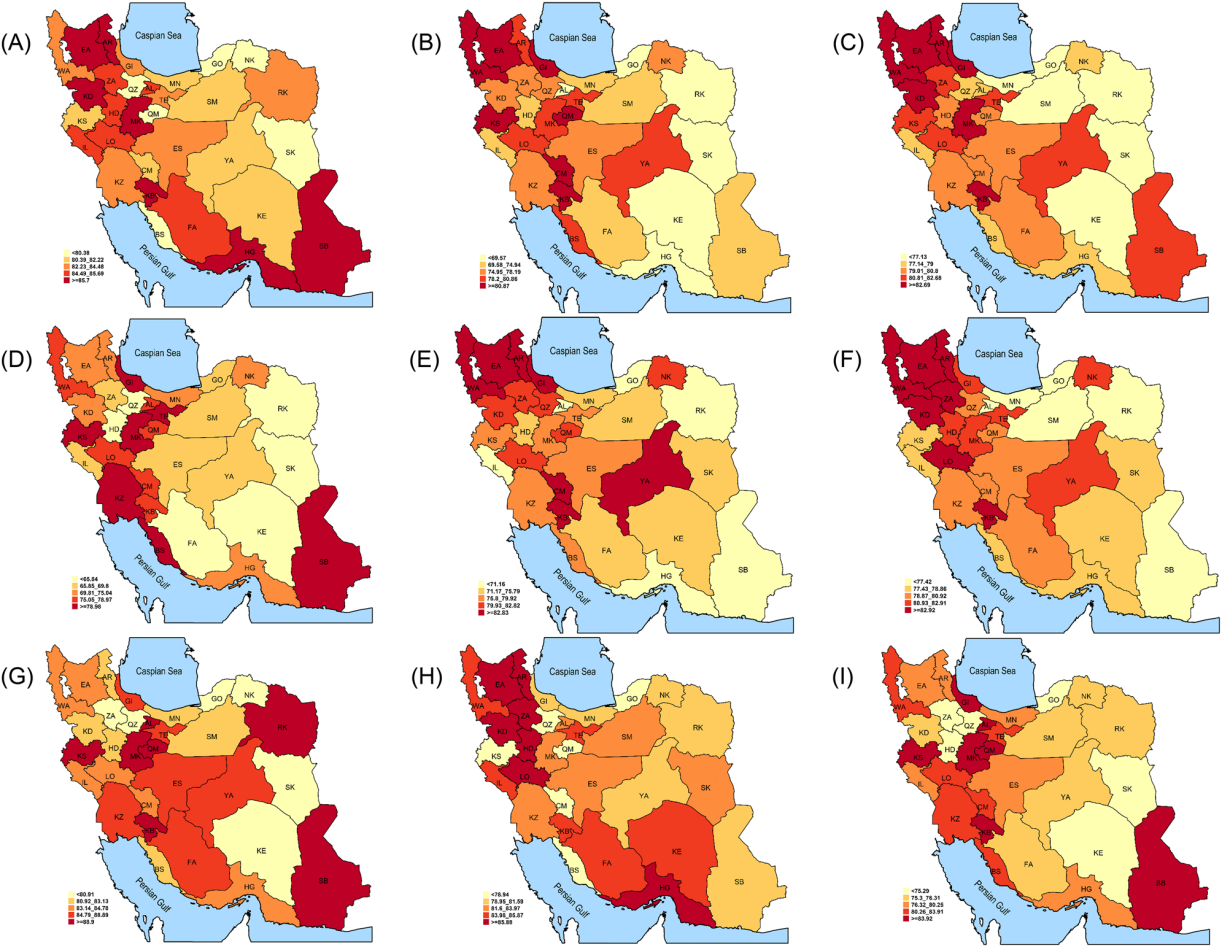
Map of Age-standardized prevalence of dyslipidemia in 2021 by residual area and sex. The figure shows the age-standardized prevalence of dyslipidemia in different subpopulation categories: (**A**) females in both rural and urban areas combined (**B**) males in both rural and urban areas combined (**C**) both sexes in both areas combined (**D**) females in urban areas (**E**) males in urban areas (**F**) both sexes in urban areas (**G**) females in rural areas (**H**) males in rural areas (**I**) both sexes in rural areas. The color scheme represents quintiles of prevalence, ranging from dark red (highest prevalence) to light yellow (lowest prevalence). The quintile assignment for each map is determined solely based on the data that encompasses the specific subpopulation described by the map. The map was drawn using R. Software version 3.2.1 (http://www.r-project.org, RRID: SCR_001905). WA indicates West Azarbayjan, WE: East Azarbayjan, AR: Ardabil, KD: Kurdistan, ZA: Zanjan, GI: Gilan, KS: Kermanshah, HD: Hamadan, QZ: Qazvin, AL: Alborz, MN: Mazandaran, GO: Golestan, IL: Ilam, LO: Lorestan, MK: Markazi, QM: Qom, TE: Tehran, SM: Semnan, NK: North Khorasan, RK: Khorasan Razavi, KZ: Khuzestan, CM: Chaharmahal and Bakhtiari, KB: Kohkiluye and Bouyerahmad, ES:Isfahan, YA: Yazd, SK: South Khorasan, BS: Boushehr, FA: Fars, KE: Kerman, SB: Sistan and Balouchestan, HG: Hormozgan.

**Figure 2:**
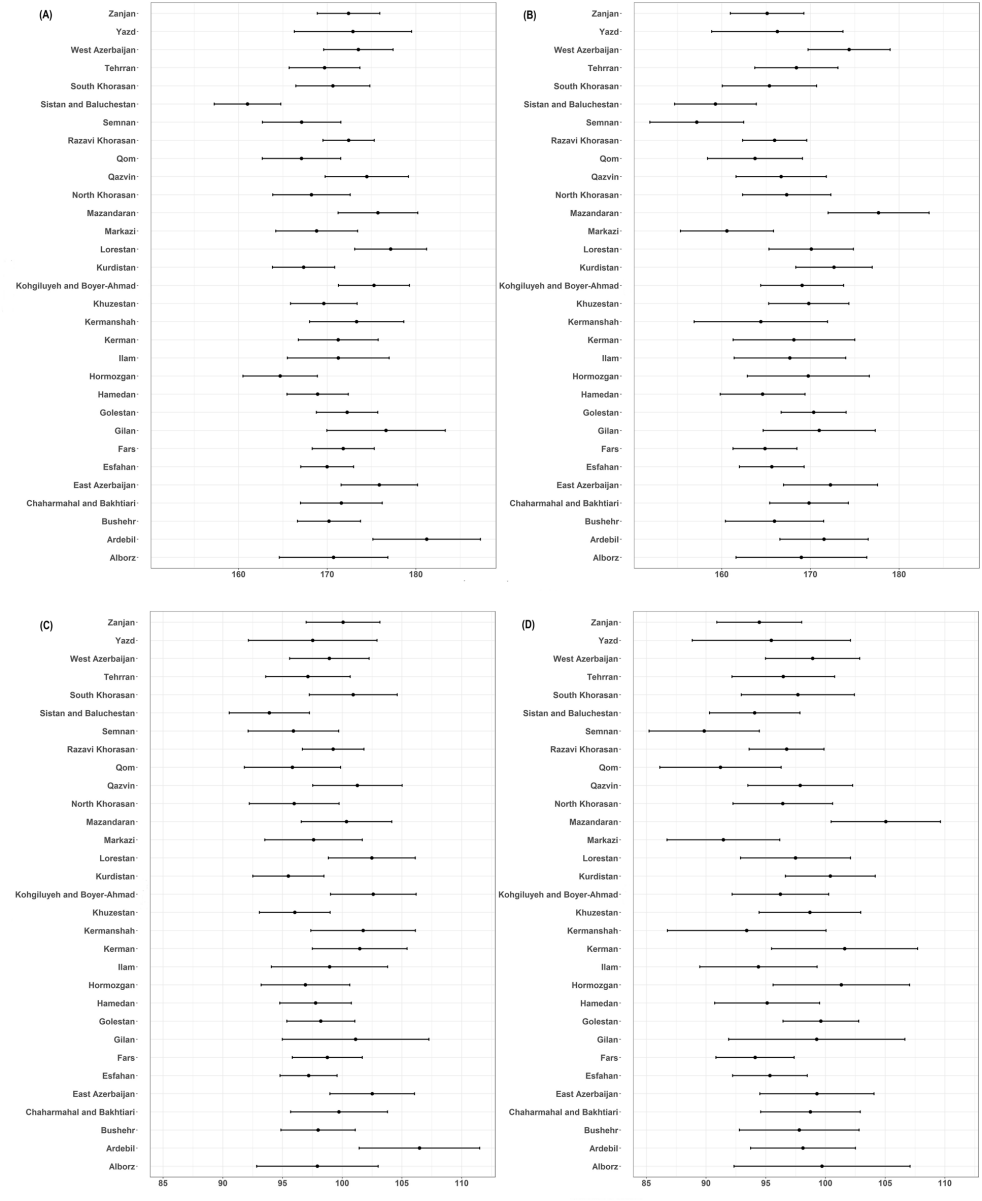
Mean Cholesterol and LDL-C in Iran’s provinces The figure shows the mean (dots) and 95% confidence interval (lines) of (**A**) female total cholesterol, (**B**) male total cholesterol, (**C**) female LDL-C, (**D**) male LDL-C, in different provinces of the country.

East Azarbaijan, Ardabil, and Kohgiluyeh and Boyer-Ahmad had the highest prevalence of dyslipidemia among provinces, with 85.3% (81.5–89.1), 84.6% (80.7–88.6), and 84.5% (80.9–88.0), respectively. On the other hand, Golestan with 68.5% (64.8–72.2), Kerman with 74.1% (69.6–78.6), and Razavi Khorasan with 74.3% (71.2–77.4) had the lowest prevalence. However, the ranking of provinces by dyslipidemia prevalence was different in specified sex groups or areas of residence (Fig. 1). The prevalence of lipid abnormalities was highly heterogeneous among provinces. Hypertriglyceridemia prevalence ranged from 50.0 to 26.7%, hypercholesterolemia prevalence ranged from 24.4 to 12.1%, high LDL-C prevalence ranged from 19.3 to 10.4%, high non-HDL-C prevalence ranged from 24.1 to 12.0, and low HDL-C prevalence ranged from 74.4 to 56.0%. Ardabil and East Azarbaijan were among the top ten provinces in the prevalence of all mentioned lipid abnormalities (high TC, TG, LDL-C, non-HDL-C, and low HDL-C). Provinces ranking in this regard was different in specified lipid abnormality and sex groups (Fig. 2). Supplementary Table S3 demonstrates the prevalence of lipid abnormalities in different provinces of the country.

## Discussion

The study showed that 81 percent of the Iranian adult population had at least one serum lipid abnormality or use lipid-lowering medications. Regardless of age, gender, or residence (rural or urban), more than 60 percent of the Iranian adult population had low HDL-C. The prevalence of dyslipidemia in the study was one of the highest across the globe; however, it was in accordance with the previous national reports with similar definition criteria for dyslipidemia, such as the STEPs 2016^10^ and the MASHAD prospective cohort study^18^. Among studies from other countries with similar definition criteria for dyslipidemia, the prevalence in healthy populations was reported at 79% in India^19^, 78.7% in Turkey^20^, 75.7% in Jordan^21^, and 62.1% in northeastern China^22^. The prevalence of low HDL-C in the studies was 72.3% in India, 41.5% in Turkey, 40.7% in Jordan, and 8.8% in northeastern China. The high prevalence of dyslipidemia in Iran can be justified by low physical inactivity, obesity, and high carbohydrate consumption^3^. Inappropriate physical activity was reported in 51.3% of the Iranian adult population^23^. Obesity also has considerably increased in recent decades, and STEPs 2021 reported overweight/obesity in 63% of the adult population^24^. This study showed obese persons are 2.8 times more at risk for dyslipidemia than normal-weight adults. This strong association and growing trend of obesity could warn the health system about the upcoming higher burden of dyslipidemia in the country. The Low HDL levels shown in the study may also be due to the high consumption of carbohydrate-rich foods, such as refined grains, which are generally inexpensive and readily available^3^. It is shown that higher carbohydrate intake, which is usually accompanied by a low fat intake, is associated with lower HDL-C and apolipoprotein A1 and higher triglycerides level^7^. In case reduced intake of trans fat has been substituted with carbohydrates rather than proteins, it would be plausible to affect HDL-C and TG negatively^10^.

On the plus side, the prevalence of hypercholesterolemia and high LDL-C was low and was seen in 21.2% and 16.4% of participants, respectively. The prevalence of hypercholesterolemia in this study was significantly lower than the rates reported in other countries, such as 48.8% in Jordan, 43% in Turkey, and 33.5% in northeastern China^20–22^. The prevalence of high LDL-C was reported at 40.7% in Jordan and 36.7% in Turkey^20, 21^. A study from Spain, which used more sensitive criteria for high LDL-C than our study, reported a 23.3% prevalence for high LDL-C^25^. Efforts taken by the government may have contributed to controlling and lowering the TC and LDL-C of the population. As of 2000, the ministry of health restricted the amount of saturated fatty acids to a maximum of 25% and trans fatty acids to a maximum of 10% in all oil products. Moreover, Iran took action to increase public awareness about the hazards of saturated and trans fatty acids. Widespread statin prescription by general practitioners was also among the country's strategies to lower serum cholesterol and prevent CVDs^10^. Iranian national action plan for NCDs includes two primary goals to be achieved by 2025: ensuring that at least 70% of eligible individuals receive drug therapy and counseling to prevent heart attacks and strokes and achieving zero trans fatty acids in food and oily products^26^.

Despite all the efforts, comparing the results of STEPs 2016 and 2021 showed that the prevalence of dyslipidemia has been unchanged from 80.1% (95% CI 79.4–80.8) in 2016 to 81% (95% CI 80.2–81.9) in 2021. In the same period, the prevalence of hypertriglyceridemia increased from 26.7% (95% CI 25.9–27.5) to 39.7% (95% CI 38.6–40.8). This shortcoming may be attributed to the mentioned obesity epidemic and changes in dietary habits. Moreover, the patients’ awareness of having dyslipidemia is shown to be as low as 20% in Iran^8^. It is indicated that the overall prevalence of undiagnosed dyslipidemia is 68.9%, while the prevalence of diagnosed dyslipidemia is only 12.5%^27^. The findings highlight a huge gap between primary and secondary care in Iran. Other reasons may be poor control of dyslipidemia despite increased medical therapy due to a lack of patients' or physicians' adherence to prescription and treatment guidelines, not optimally-dosed statin prescriptions, and not using combination therapies when necessary^28^. The COVID-19 epidemic may also impact the control of serum cholesterol worldwide^29–31^. Due to the curtailment in routine outpatient laboratory testing, lipid-lowering medical therapy has been delayed, and drug shortages and misinformation may compromise adherence to these medications. Furthermore, the mobilization of the health workforce to combat COVID-19 could limit access to health care. From a societal perspective, the unprecedented contraction of social and economic activities has led to social isolation and decreased physical activity.

Dyslipidemia was more prevalent in northwestern parts of the country (Fig. 1). Iran is a country with great heterogeneity in cultural heritage and ethnicity. From a cultural view, it is shown that undergoing urbanization and westernization and having a food culture providing a higher uptake of calories are associated with dyslipidemia. Ethnic diversity could also translate into substantial variation in the prevalence of dyslipidemia and plasma lipid levels between and within countries. Ethnicity also can affect response to statin therapy, which is related to genetic differences in the metabolism of statins^32^. However, research about such geographical differences is scarce in Iran, and the object needs further studies.

Some of the study results highlighted the need for cautious interpretation, particularly where associations are interpreted between dyslipidemia and specific medical conditions such as the history of cardiovascular diseases, diabetes, and stroke in which lipid-lowering medications are more often indicated. In this study, it is observed that participants with a history of coronary heart disease had a higher prevalence of dyslipidemia; however, they showed a lower prevalence of hypercholesterolemia, high LDL-C, and mixed dyslipidemia. The result could be attributed to the higher rate of consuming lipid-lowering medications in this group, which can effectively manage the TC and LDL-C levels of the patients. However, these individuals are still categorized as having dyslipidemia (by definitions of the study); therefore, the group can have a higher prevalence of dyslipidemia despite having a lower high TC and LDL-C prevalence. This justification could be also the case for not showing a positive association between mixed dyslipidemia and diabetes or a history of stroke.

The study also showed no association between dyslipidemia and wealth index. Although those with lower income usually get lower amounts of calories, their regimen contains very high amounts of carbohydrates, especially from refined sources (such as white rice and white bread), which is associated with higher odds of having hypercholesterolemia, hypertriglyceridemia, and low HDL-C^3, 7, 28^. Therefore, the effect of wealth and poverty on the lipid profile is influenced by many counter-acting factors, and further studies are needed in this regard. Furthermore, our study results indicated no significant associations between dyslipidemia and fruit and vegetable consumption or smoking, consistent with previous findings from STEPs 2016^10^. The impact of fruit and vegetable intake on the lipid profile has remained inconclusive in the literature^33^. Some studies show a diet high in fruits and vegetables does not improve lipid profile, while other studies show significant improvement in lipid profile after consuming a diet high in fruits and vegetables^34, 35^. There might be a need for further adjustment of the models for confounding variables such as body mass index, energy intake, smoking status, dietary cholesterol, and history of diabetes mellitus and coronary artery disease to better assess the association of fruit and vegetable consumption on the lipid profile^33^. Regarding the association between smoking and dyslipidemia, although we showed no association between the prevalence of dyslipidemia and smoking status, higher serum levels of triglyceride and lower HDL-C serum level was observed in the smoker group (Supplementary Table 2). There is solid evidence of the role of smoking in deteriorating the levels of plasma lipids. Studies suggest that nicotine stimulates the secretion of catecholamines, cortisol, and growth hormones, which increase the serum-free fatty acid concentration and further stimulate the secretion of very low-density lipoproteins and triglycerides in the liver^36^. We believe that the higher prevalence of smoking among men than women (26.0% vs. 4.6%) and the reverse association between being men and dyslipidemia (OR = 0.56) was the major confounding factor in the result. As it is shown by the study, the odds of having dyslipidemia in the smoker group increased from 0.88 to 1.14 after adjusting groups for sex, age, and wealth index. Other possible confounding factors such as possible differences in consuming lipid-lowering medications between the groups should be considered in future studies.

The STEPs 2021 is the second population-based STEPs survey in Iran that represented a complete lipid profile of the Iranian adult population and is the first one that covers all provinces of the country in this regard. As a result of the COVID-19 pandemic specifications, the design and implementation of the survey with special considerations for COVID-19 protection and safety is the most significant achievement of this STEPs survey in Iran. The findings of our study, however, should be considered with an understanding of the limitation that dyslipidemia and other lipid abnormalities are defined differently in different studies; and it is important to carefully compare our variable definitions with those of others.

Poor metabolic health is a major concern in Iran, which is now a country with a population exceeding 80 million, mostly living in urban regions. The substantial increase in the prevalence of overweight/obesity and especially among adolescents might soon lead to larger increases in diabetes and dyslipidemia^37^. Therefore, controlling dyslipidemia would be a milestone to maintain the declining rate of premature deaths due to cardiovascular diseases in the country and achieve a one-third reduction in mortality from NCDs by 2030^38^. In conclusion, the current nationwide study showed that although the prevalence of high TC and LDL-C have a favorable trend, the prevalence remained high in the case of low HDL-C and aggravated in the case of high TG. Altogether, the prevalence of dyslipidemia remained unchanged from 2016 to 2021.

The present study could have profound implications. The confirmation that current ongoing programs and interventions are insufficient to decrease dyslipidemia prevalence highlights the urgency for more effective and targeted approaches. The study findings help policymakers and health executives to obtain a more accurate estimation of the dyslipidemia problem and to implement more effective interventional programs. The identification of modifiable risk factors that contribute to dyslipidemia emphasizes the need for comprehensive lifestyle interventions. The study results highlight the urgent need to modify the overweight/obesity trend as the condition with the highest association with dyslipidemia and 63% prevalence in the over 18 years-old Iranian population^24^. There are effective and cost-efficient interventions recommended by WHO for the prevention and control of NCDs and modifying dietary risk factors of obesity and dyslipidemia. The interventions include eliminating industrial trans-fats through the development of legislation, reducing sugar consumption through effective taxation on sugar-sweetened beverages, granting subsidies to increase the intake of fruits and vegetables, replacing trans and saturated fats with unsaturated ones, limiting portion and package size of products to reduce energy intake, implementing nutrition education and counseling in different settings, and launching mass media campaigns on healthy diets^23^. As another modifiable risk factor, physical inactivity prevalence in Iran is shown to be 41.9% in men and 57.9% in females in 2021^23^. The factors discouraging women from being physically active in the cultural context of Iran should be mitigated. These factors could be personal issues (no motivation, enjoyment, or skills in sport), lack of social support, environmental barriers (not enough free time or access to sports facilities), and cultural stigma^3^. Furthermore, the study reveals the importance of targeting high-risk groups, such as patients with diabetes and hypertension, with more focused public education and screening initiatives. Altogether, modifying NCD risk factors and promoting metabolic health in the country require action plans to come to action through a multi-sectoral and collaborative approach. Although action plans to achieve global and national level goals have been developed in Iran^26^, there is a lack of tracking systems to show whether we stay forward or lag behind to achieve the goals. We believe that the present study serves as a crucial step in addressing this gap by providing up-to-date data on the prevalence of dyslipidemia, as a major NCD risk factor in the Iranian adult population.

### Supplementary Information

Supplementary Table 1.

Supplementary Table 2.

Supplementary Table 3.

## Data Availability

The datasets used and/or analysed during the current study available from the corresponding author on reasonable request.
