# Supplementary material for: Prevalence of plasma lipid abnormalities and associated risk factors among Iranian adults based on the findings from STEPs survey 2021
Source: Sci Rep. 2023 Sep 19;13:15499. doi: 10.1038/s41598-023-42341-5 (PMC10509214; doi:10.1038/s41598-023-42341-5)
Supplement: Supplementary file 1 — Supplementary Table 1. [file 41598_2023_42341_MOESM1_ESM.pdf]

Supplementary Table 1: Serum lipid levels for Iranian adults, 2021							
Age	Number of individuals	Mean	Selected percentiles				
			5th	25th	50th	75th	95th
Serum triglyceride (mg/dl)							
Women	10281						
25-35	2,074	122.09 (117.45,126.73)	52.20	75.00	105.00	146.0	264.00
35-45	2,647	137.82 (133.09,142.56)	58.00	85.00	117.00	166.0	289.00
45-55	2,403	151.98 (147.85,156.1)	67.00	101.00	134.00	183.2	293.20
55-65	1,807	160.71 (155.83,165.59)	75.00	108.00	144.50	191.0	309.00
65-75	1,034	154.01 (147.63,160.4)	77.00	105.00	136.00	180.0	290.00
>75	316	155.5 (138.39,172.6)	69.70	105.00	140.00	185.0	269.00
Men	7814						
25-35	1,469	158.16 (149.02,167.31)	60.00	92.00	131.00	187.0	371.00
35-45	1,812	168.73 (162.92,174.54)	64.00	107.00	147.00	205.6	344.00
45-55	1,689	176.6 (169.15,184.05)	68.00	106.00	149.00	209.0	380.00
55-65	1,499	154.61 (149.6,159.62)	67.00	99.00	138.00	188.6	309.00
65-75	922	146.28 (138.92,153.64)	65.00	94.00	128.00	180.0	274.00
>75	423	130.95 (123.03,138.87)	65.80	90.00	121.00	159.0	246.00
Serum total cholesterol (mg/dl)							
Women	10281						
25-35	2,074	159.9 (158.01,161.8)	116.00	138.00	156.00	177.0	215.00
35-45	2,647	168.05 (166.44,169.66)	122.00	146.50	165.00	187.0	223.00
45-55	2,403	181.98 (179.93,184.03)	127.00	156.00	179.00	204.0	247.00
55-65	1,807	185.34 (182.79,187.9)	121.80	157.00	185.00	212.0	252.00
65-75	1,034	179.82 (176.78,182.85)	120.00	153.20	176.40	205.0	248.00
>75	316	184.61 (171.42,197.81)	120.00	153.00	179.70	212.0	266.50
Men	7816						

25-35	1,469	161.77 (159.56,163.98)	115.00	139.00	159.30	182.0	221.00
35-45	1,813	172.86 (170.16,175.57)	121.20	149.00	169.00	193.0	233.00
45-55	1,690	174.16 (171.92,176.4)	120.00	150.00	173.00	198.0	235.00
55-65	1,499	170.14 (167.34,172.94)	106.00	145.00	169.00	194.0	234.00
65-75	922	166.1 (161.51,170.68)	105.00	137.00	164.00	189.0	239.00
>75	423	161.9 (156.5,167.31)	93.00	129.00	164.40	186.0	225.00
Serum LDL-C (mg/dl)							
Women	10278						
25-35	2,074	91.17 (89.51,92.84)	53.70	73.46	88.80	105.8	136.00
35-45	2,645	96.49 (95.1,97.87)	54.30	78.80	94.90	112.2	142.70
45-55	2,403	106.02 (104.27,107.77)	57.60	83.40	104.90	126.3	161.20
55-65	1,806	107.42 (105.19,109.64)	51.30	81.30	107.22	131.3	165.80
65-75	1,034	102.57 (99.83,105.32)	49.34	76.20	102.30	125.8	161.28
>75	316	108.16 (97.36,118.96)	52.70	78.70	107.80	133.9	173.18
Men	7807						
25-35	1,467	91.56 (89.55,93.58)	49.20	71.10	89.00	109.8	141.70
35-45	1,809	101.44 (98.95,103.93)	55.90	81.24	98.90	118.9	152.30
45-55	1,687	100.97 (98.81,103.13)	48.00	79.40	100.40	122.2	154.20
55-65	1,499	99.92 (97.53,102.3)	43.80	77.70	99.30	122.3	156.30
65-75	922	97.2 (93.41,100.99)	43.70	71.00	95.30	119.5	161.00
>75	423	95.51 (90.96,100.07)	36.10	69.70	96.96	114.9	151.80
Serum HDL-C (mg/dl)							
Women	10279						
25-35	2,073	44.33 (43.76,44.89)	30.30	37.00	43.00	49.5	63.40
35-45	2,647	44.04 (43.5,44.58)	30.00	36.90	42.90	49.8	62.00

45-55	2,402	45.56 (45.01,46.11)	31.20	38.40	43.90	51.0	65.20
55-65	1,807	45.89 (45.24,46.55)	30.90	37.90	45.10	52.0	64.80
65-75	1,034	46.44 (45.42,47.47)	31.20	38.30	44.90	53.0	68.70
>75	316	45.35 (43.94,46.76)	31.10	37.90	43.30	51.1	62.50
Men	7813						
25-35	1,469	26.80	32.40	37.20	43.5	57.00	26.80
35-45	1,812	26.60	31.80	36.70	42.2	53.70	26.60
45-55	1,688	26.20	32.30	37.40	42.6	52.60	26.20
55-65	1,499	27.00	33.20	37.40	44.3	56.60	27.00
65-75	922	27.30	32.80	38.60	44.2	55.10	27.30
>75	423	27.50	33.80	39.40	45.6	56.20	27.50
