# Supplementary material for: Prevalence of plasma lipid abnormalities and associated risk factors among Iranian adults based on the findings from STEPs survey 2021
Source: Sci Rep. 2023 Sep 19;13:15499. doi: 10.1038/s41598-023-42341-5 (PMC10509214; doi:10.1038/s41598-023-42341-5)
Supplement: Supplementary file 2 — Supplementary Table 2. [file 41598_2023_42341_MOESM2_ESM.pdf]

Supplementary Table 2: Mean triglyceride, cholesterol, LDL-C, non-HDL-C, and HDL-C according to study population strata						
Variable	Category	Triglyceride (mg/dl), mean (95% CI)	Total cholesterol (mg/dl), mean (95% CI)	LDL-C (mg/dl), mean (95% CI)	non–HDL-C (mg/dl), mean (95% CI)	HDL-C (mg/dl), mean (95% CI)
Sex	Women	144.15 (141.95,146.35)	174.38 (173.3,175.45)	100.52 (99.61,101.42)	129.33 (128.25,130.4)	45.06 (44.79,45.34)
	Men	160.92 (157.84,164)	169 (167.79,170.21)	98.25 (97.16,99.34)	130.34 (129.1,131.57)	38.67 (38.4,38.94)
Age	25-39	141.17 (137.56,144.77)	163.03 (161.91,164.15)	93.19 (92.19,94.2)	121.34 (120.17,122.51)	41.7 (41.36,42.05)
	40-54	160.06 (156.81,163.3)	176.87 (175.58,178.16)	102.9 (101.74,104.07)	134.87 (133.54,136.2)	42.02 (41.67,42.37)
	55-64	157.94 (154.43,161.44)	178.43 (176.49,180.38)	104 (102.36,105.65)	135.53 (133.68,137.39)	42.9 (42.39,43.4)
	>65	147.91 (143.58,152.24)	172.94 (170.14,175.75)	100.34 (98.02,102.66)	129.92 (127.12,132.71)	43.02 (42.45,43.59)
Area	Rural	145.5 (142.84,148.17)	172.38 (171.26,173.51)	100.9 (99.93,101.86)	129.93 (128.8,131.05)	42.47 (42.16,42.79)
	Urban	153.58 (151.3,155.87)	171.86 (170.86,172.87)	99.06 (98.18,99.93)	129.72 (128.71,130.73)	42.15 (41.89,42.4)
Wealth index	Poor	145.86 (141.95,149.78)	172.3 (170.09,174.52)	100.63 (98.79,102.47)	129.68 (127.48,131.89)	42.62 (42.18,43.06)
	2nd quintile	150.55 (146.56,154.54)	170.66 (169.05,172.28)	98.36 (96.95,99.78)	128.49 (126.85,130.13)	42.2 (41.7,42.7)
	Middle	150.91 (147.2,154.63)	170.51 (168.95,172.06)	98.72 (97.35,100.08)	128.84 (127.3,130.37)	41.7 (41.28,42.12)
	4th quintile	155.1 (151.16,159.04)	172.3 (170.79,173.81)	99.21 (97.91,100.51)	130.21 (128.7,131.72)	42.09 (41.66,42.52)
	Rich	156.44 (151.05,161.83)	173.85 (171.66,176.03)	100.53 (98.57,102.48)	131.73 (129.49,133.96)	42.12 (41.56,42.69)
BMI Category	Underweight	95.79 (92.03,99.55)	155.95 (152.52,159.38)	88.44 (85.26,91.63)	107.6 (104.15,111.06)	48.35 (47.02,49.67)
	Normal weight	130.27 (127.42,133.13)	167.31 (165.93,168.68)	97.54 (96.39,98.69)	123.53 (122.16,124.91)	43.77 (43.39,44.16)
	Overweight	156.85 (154.04,159.66)	173.8 (172.55,175.04)	101.16 (100.05,102.28)	132.48 (131.24,133.72)	41.33 (41,41.65)
	Obese	172.43 (168.35,176.51)	175.54 (173.87,177.22)	99.85 (98.39,101.31)	134.29 (132.61,135.97)	41.28 (40.9,41.65)
Fruit and Vegetable Consumption	Inappropriate	151.34 (149.41,153.26)	171.93 (171.1,172.77)	99.49 (98.76,100.22)	129.72 (128.88,130.57)	42.22 (42.01,42.44)
	Appropriate	155.83 (149.34,162.32)	172.54 (169.68,175.41)	99.43 (96.98,101.89)	130.26 (127.48,133.03)	42.29 (41.4,43.18)
Physical activity	Appropriate	152.48 (149.43,155.53)	172.05 (170.92,173.18)	99.53 (98.54,100.52)	129.99 (128.86,131.12)	42.06 (41.76,42.36)
	Inappropriate	152.12 (149.67,154.57)	172.42 (171.13,173.71)	99.47 (98.36,100.58)	129.84 (128.54,131.14)	42.6 (42.28,42.91)
Smoking	No	149.59 (147.67,151.5)	172.43 (171.58,173.27)	99.75 (99.03,100.48)	129.62 (128.77,130.47)	42.81 (42.59,43.03)
	Yes	163.78 (157.9,169.66)	169.13 (166.69,171.58)	97.85 (95.62,100.07)	130.53 (128.02,133.04)	38.64 (38.12,39.16)
Diabetes	No	145.55 (143.69,147.4)	172.7 (171.86,173.54)	101.08 (100.35,101.81)	130.14 (129.29,130.99)	42.58 (42.35,42.8)
	Yes	188.34 (181.97,194.72)	167.51 (165.04,169.99)	89.81 (87.8,91.82)	127.41 (124.97,129.86)	40.1 (39.58,40.62)
Hypertension	No	143.48 (141.36,145.6)	170.13 (169.19,171.07)	98.96 (98.13,99.78)	127.59 (126.64,128.55)	42.54 (42.28,42.8)
	Yes	166.01 (162.58,169.43)	175.12 (173.63,176.6)	100.33 (99.04,101.61)	133.48 (132.02,134.95)	41.66 (41.33,41.99)
History of coronary heart disease	No	151.19 (149.27,153.1)	173.11 (172.29,173.93)	100.58 (99.86,101.29)	130.75 (129.92,131.59)	42.37 (42.15,42.59)
	Yes	156.36 (149.4,163.32)	158.09 (154.9,161.28)	86.3 (83.65,88.96)	117.58 (114.49,120.66)	40.51 (39.79,41.24)
History of stroke	No	151.54 (149.67,153.4)	172.07 (171.26,172.88)	99.59 (98.88,100.29)	129.84 (129.02,130.66)	42.24 (42.03,42.45)

	Yes	154.91 (142.67,167.15)	163.65 (157.41,169.89)	91.41 (85.79,97.03)	122.39 (116.25,128.53)	41.27 (39.72,42.81)
Familial History of CVD	No	153.62 (148.09,159.16)	172.12 (169.55,174.68)	98.96 (96.66,101.26)	129.69 (127.13,132.24)	42.43 (41.63,43.23)
	Yes	152.57 (150.46,154.67)	172.01 (171.1,172.93)	99.54 (98.75,100.33)	129.99 (129.07,130.9)	42.04 (41.82,42.27)
