# Supplementary material for: Prevalence of plasma lipid abnormalities and associated risk factors among Iranian adults based on the findings from STEPs survey 2021
Source: Sci Rep. 2023 Sep 19;13:15499. doi: 10.1038/s41598-023-42341-5 (PMC10509214; doi:10.1038/s41598-023-42341-5)
Supplement: Supplementary file 3 — Supplementary Table 3. [file 41598_2023_42341_MOESM3_ESM.pdf]

Supplementary Table 3: Serum Lipid Status in Different Provinces of Iran								
Province	Population based on census 2016 (1000 people)	Hypertriglyceridemia Percent. (95% CI)	Hypercholesterolemia Percent. (95% CI)	High LDL-C Percent. (95% CI)	Very High LDL-C Percent. (95% CI)	High non–HDL-C Percent. (95% CI)	Low HDL-C Percent. (95% CI)	Dyslipidemia Percent. (95% CI)
Alborz	2712	37.77 (30.34,45.2)	18.09 (13.1,23.08)	12.71 (8.37,17.06)	0.41 (-0.17,1)	15.46 (10.64,20.27)	68.72 (62.15,75.29)	77.23 (70.87,83.58)
Ardabil	1270	49.98 (44.49,55.46)	24.42 (19.85,28.99)	16.74 (12.98,20.5)	1.41 (0.2,2.62)	24.09 (19.43,28.74)	74.13 (69.43,78.84)	84.64 (80.72,88.57)
Azerbaijan, East	3909	45.01 (39.53,50.49)	22.26 (18.39,26.13)	17.26 (13.53,20.99)	0.93 (0.21,1.66)	21.68 (17.76,25.61)	74.4 (69.88,78.92)	85.32 (81.55,89.1)
Azerbaijan, West	3265	47.63 (43.05,52.22)	21.35 (17.95,24.75)	14.79 (11.77,17.8)	0.59 (-0.09,1.28)	19.35 (16.1,22.6)	71.65 (67.56,75.74)	83.08 (79.66,86.5)
Bushehr	1163	35.31 (31.12,39.51)	18.64 (15.4,21.88)	15.63 (12.58,18.68)	0.84 (-0.19,1.87)	16.2 (13.12,19.29)	68.39 (64.14,72.64)	78.77 (74.9,82.63)
Chahar Mahaal and Bakhtiari	947	38.34 (33.96,42.72)	20.14 (16.73,23.55)	15.75 (12.7,18.8)	1.4 (0.18,2.63)	19.08 (15.73,22.44)	69.53 (65.36,73.71)	80.27 (76.68,83.86)
Fars	4851	35.03 (31.54,38.52)	18.17 (15.55,20.79)	14.19 (11.79,16.59)	0.6 (0.09,1.1)	16.31 (13.79,18.84)	68.17 (64.68,71.66)	79.5 (76.39,82.6)
Gilan	2530	42.47 (35.54,49.4)	23.18 (17.62,28.74)	17.27 (12.27,22.27)	0.37 (0,0.74)	21.43 (16.1,26.76)	71.55 (65.77,77.33)	83.45 (78.44,88.47)
Golestan	1868	30.57 (26.93,34.21)	18.74 (15.79,21.68)	13.54 (10.96,16.11)	0.48 (0.04,0.92)	16.81 (13.92,19.69)	56.01 (52.08,59.95)	68.5 (64.83,72.17)
Hamadan	1758	31.58 (27.27,35.88)	17.93 (14.73,21.12)	13.24 (10.37,16.1)	0.17 (-0.07,0.42)	16.51 (13.38,19.64)	69.62 (65.22,74.02)	79.01 (74.98,83.04)
Hormozgan	1776	27.53 (22.47,32.59)	13.41 (9.79,17.03)	10.42 (7.25,13.59)	0.42 (-0.16,0.99)	12.96 (9.35,16.58)	73.36 (68.57,78.14)	78.88 (74.42,83.35)
Ilam	580	39.22 (33.67,44.77)	21.24 (16.92,25.56)	13.84 (10.26,17.42)	1.11 (0.06,2.16)	17.73 (13.67,21.79)	65.02 (59.49,70.54)	77.26 (72.31,82.21)
Isfahan	5120	37.27 (33.71,40.84)	18.1 (15.59,20.6)	12.52 (10.4,14.65)	0.48 (0,0.97)	16.26 (13.77,18.75)	67.79 (64.52,71.06)	79.9 (77.04,82.76)
Kerman	3164	28.06 (23.26,32.86)	20.74 (16.28,25.2)	19.33 (14.95,23.71)	1.01 (0.24,1.79)	19.65 (15.25,24.06)	64.83 (59.92,69.74)	74.11 (69.6,78.62)
Kermanshah	2530	37.42 (31.55,43.29)	16.54 (10.78,22.29)	11.48 (8.09,14.86)	0.53 (-0.02,1.08)	14.22 (10.57,17.87)	72.15 (66.17,78.14)	81.03 (75.48,86.58)
Khorasan, North	863	35.1 (30.98,39.23)	19.58 (16.32,22.84)	13.88 (11.17,16.59)	0.61 (0.07,1.14)	17.7 (14.59,20.81)	65.58 (61.49,69.66)	78.86 (75.35,82.38)
Khorasan, Razavi	6434	30.12 (27.07,33.17)	19.05 (16.57,21.53)	15.35 (13.07,17.62)	0.32 (0.04,0.61)	17.33 (14.83,19.82)	59.7 (56.26,63.14)	74.31 (71.18,77.44)
Khorasan, South	768	26.7 (22.66,30.75)	17.12 (13.96,20.28)	15.43 (12.34,18.51)	0.56 (-0.02,1.14)	16.08 (12.97,19.19)	65.45 (60.81,70.1)	75.07 (70.69,79.45)
Khuzestan	4710	40.22 (35.83,44.6)	17.57 (14.49,20.66)	12.79 (9.99,15.58)	0 (0,0)	16.11 (13.07,19.15)	70.45 (66.49,74.41)	80.12 (76.54,83.7)
Kohgiluyeh and Boyer-Ahmad	713	43.71 (39.18,48.24)	19.44 (15.97,22.9)	15.13 (12.1,18.16)	0.47 (-0.06,0.99)	17.89 (14.57,21.21)	74.37 (70.25,78.49)	84.47 (80.95,87.98)
Kurdistan	1603	41.15 (36.94,45.35)	16.52 (13.47,19.57)	12.95 (10.24,15.65)	0.48 (0,0.95)	17.26 (14.16,20.35)	72.81 (69,76.62)	83.26 (80,86.51)
Lorestan	1760	42.45 (38.11,46.78)	22.39 (18.77,26.02)	16.43 (13.26,19.61)	0.82 (0.03,1.62)	19.05 (15.61,22.48)	70.86 (66.74,74.98)	82.05 (78.46,85.63)
Markazi	1429	33.69 (28.33,39.06)	16.27 (12.8,19.74)	12.02 (9.05,14.99)	0.35 (-0.05,0.74)	13.14 (10.01,16.27)	71.33 (66.35,76.31)	82.69 (78.29,87.09)
Mazandaran	3283	40.12 (35.11,45.14)	23.51 (19.15,27.87)	16.85 (13.02,20.68)	1.5 (0.46,2.54)	23.2 (18.76,27.64)	59.1 (53.91,64.29)	76.62 (71.89,81.36)
Qazvin	1273	34.58 (29.45,39.72)	18.5 (14.29,22.71)	14.55 (10.92,18.19)	0.12 (-0.11,0.35)	17.83 (13.56,22.11)	64.18 (58.82,69.53)	77.14 (72.32,81.96)
Qom	1292	37.88 (31.91,43.85)	17.26 (13.48,21.04)	12.02 (8.88,15.17)	0.18 (-0.17,0.53)	16.26 (12.58,19.95)	71.64 (66.57,76.71)	79.21 (74.52,83.9)
Semnan	702	29.94 (25.04,34.83)	13.87 (10.49,17.26)	11.98 (8.75,15.2)	0.74 (-0.04,1.51)	12.4 (9.1,15.7)	66.58 (61.21,71.95)	76.82 (71.86,81.78)
Sistan and Baluchistan	2775	27.42 (23.48,31.35)	12.08 (9.37,14.8)	12.34 (9.6,15.09)	0.3 (-0.15,0.76)	11.99 (9.3,14.67)	73.18 (69.01,77.35)	80.85 (76.96,84.73)
Tehran	13267	39.43 (34.99,43.88)	18.16 (14.84,21.48)	15.78 (12.36,19.21)	0.79 (-0.07,1.65)	16.78 (13.58,19.98)	73.11 (69.63,76.59)	81.95 (78.85,85.05)
Yazd	1138	45.89 (39.28,52.5)	21.48 (16.34,26.61)	13.56 (9.52,17.59)	2.09 (0.49,3.69)	19.14 (14.22,24.06)	71.94 (66.38,77.5)	80.81 (75.93,85.68)

Zanjan	1057	39.57 (35.79,43.34)	17.39 (14.63,20.15)	13.61 (11.12,16.1)	0.5 (-0.02,1.03)	17.48 (14.69,20.27)	73 (69.63,76.37)	80.9 (77.87,83.94)
